# Association between Self-Reported Survey Measures and Biomarkers of Second-Hand Tobacco Smoke Exposure in Non-Smoking Pregnant Women

**DOI:** 10.3390/ijerph18179197

**Published:** 2021-08-31

**Authors:** Meiman Maggie Chen, Su-Er Guo, Chi-Pin Yuan, Chizimuzo Okoli, Yen-Chi Liao

**Affiliations:** 1 Department of Nursing and Graduate Institute of Nursing, College of Nursing, Chang Gung University of Science and Technology (CGUST), Puzi City 613016, Taiwan; mmchen@mail.cgust.edu.tw (M.M.C.); seguo@mail.cgust.edu.tw (S.-E.G.); 2 Chronic Diseases and Health Promotion Research Center, Chang Gung University of Science and Technology (CGUST), Puzi City 613016, Taiwan; 3 Division of Pulmonary and Critical Care Medicine, Chang Gung Memorial Hospital, Puzi City 613016, Taiwan; 4 Department of Safety Health and Environmental Engineering, Ming Chi University of Technology, New Taipei City 243303, Taiwan; 5 Department of Nursing, Ditmanson Medical Foundation Chiayi Christian Hospital, Chiayi City 600566, Taiwan; 01454@cych.org.tw; 6 Behavioral Health Wellness Environments for Living and Learning (BH WELL), College of Nursing, University of Kentucky, 315 College of Nursing Building, Lexington, KY 40536, USA; ctokol1@uky.edu

**Keywords:** biomarkers, environmental tobacco smoke exposure, pregnant women, urine cotinine

## Abstract

Second-hand tobacco smoke (SHS) causes adverse health outcomes in adults. Further studies are needed to evaluate psychosocial SHS exposure measures in comparison to SHS exposure biomarkers, particularly in pregnant women. This study aimed to compare self-reported SHS exposure to urinary cotinine levels in pregnant women. A cross-sectional correlation design was conducted using a convenience sample of 70 non-smoking pregnant women. Measures included self-reported questionnaires and laboratory confirmation of cotinine levels in the urinary samples. Multiple regression analysis was used to assess the correlation after controlling for potential confounding variables. The average level of urinary cotinine among non-smoking pregnant women was 6.77 ng/mL. Medium-strength correlations were found among psychosocial SHS exposure measures and urine cotinine levels. Questions regarding ‘instances of smoking in front of the individual’ and ‘subjective perceived frequency of SHS exposure in past 7 days’ are feasible items for pregnant women in clinics (particularly the first question). Hence, we suggest that these simple questions should be used to assist pregnant women in reducing the harm associated with SHS exposure.

## 1. Introduction

Despite global efforts to reduce active smoking, women and children are still exposed to second-hand tobacco smoke (SHS) at home and the workplace. Studies from Spain, Taiwan, and China revealed staggering rates of SHS exposure in non-smoking women. The rate of SHS exposure has been reported to be 63.1% among non-smoking women in Spain [1]. In Taiwan, SHS exposure among non-smoking pregnant women because of their spouse’s smoking has been reported to be 46% [2]. In China, among 3165 non-smoking households, 87% of the respondents reported that smokers would smoke in front of them [3].

Studies have shown that SHS exposure causes premature death in children and adults [4]. The harmful effects of SHS exposure may be increased in pregnant women because of its effects on the developing organs in the foetus. In addition, prenatal SHS exposure is associated with adverse effects on child health [5]. The adverse pregnancy and birth outcomes include small for gestational age [6], foetal growth restriction [7,8,9,10], poor respiratory health during the first year of life [1], and stillbirth [11].

Reliable health information for SHS detection is a key component for the formulation of health policies and education programmes. Studies evaluating self-reported SHS exposure to different biomarkers among public health and maternal populations have reported mainly using self-reported methods [12,13,14]. Previous public health studies used SHS exposure self-reported questionnaires to compare airborne nicotine levels [15] and different cotinine biomarkers [16]. These two studies found a moderate association between self-reported SHS questionnaire scores of an adult population and airborne nicotine and cotinine biomarkers. 

However, in non-smoking pregnant women, self-reported SHS exposure may be underestimated. Xiao et al.’s study included 420 Chinese pregnant women residing in two rural towns to compare self-reported SHS exposure to urinary cotinine levels [17]. Self-reported SHS exposure was obtained through face-to-face interviews and cotinine levels were determined using urine samples. The self-reported rate of SHS was 76.9%, 83.3%, and 80.5% in the first, second, and third trimesters, respectively. The rate of urinary cotinine-verified (UC-verified) SHS was determined using the limit of detection at 0.08 ng/mL by high-performance liquid chromatography/tandem spectrometry. The self-reported rate of SHS was 76.9%, 83.3%, and 80.5% in the first, second, and third trimesters, respectively, whereas the rate of UC-verified SHS was 97.4%, 99.1%, and 98.6% in the first, second, and third trimesters, respectively. The gradient differences between SHS exposure and urine cotinine levels were 25.2%, 17.1%, and 20.5% in the first, second, and third trimesters, respectively. It seems that self-reported SHS exposure is underestimated in rural Chinese women partly due to methods used to determined urinary cotinine [17]. However, it is unknown whether Chinese culture may influence pregnant women with self-reported SHS exposure. 

In the United States, Gavarkovs et al. [18] examined the consistency of SHS exposure among two SHS self-reported measurements and salivary cotinine in 147 non-smoking American women at 32 weeks’ gestation and 6 months postpartum. The first self-reported measurement contained five different sites in the SHS exposure questionnaire. The second self-reported SHS exposure used a validated scale of the ‘Environment Tobacco Smoke Avoidance’ questionnaire. Higher values indicate greater avoidance of the SHS. Gavarkovs et al. found that the SHS avoidance score was inversely associated with the number of SHS exposure sites [18]. For example, the avoidance score (2.9) in two or more settings was lower than one site (3.2 < 0.05). This finding suggests that proactive avoidance may be a reliable method for detecting SHS exposure. Similarly, higher saliva cotinine with a higher number of reported exposure locations suggested that the inclusion of more exposure settings into questionnaire items may be favourable. Therefore, it is important to use avoidance as an alternative to measuring SHS exposure. 

Evaluating self-reported SHS exposure is less costly and non-invasive. However, the quality of self-reported SHS exposure results may be influenced by recall errors, misclassification, the collection method, and social norms of smoking behaviour [5]. Chiang and Chang [19] found that the rate of self-administered SHS exposure was 2–3% lower than the rate of self-reported SHS exposure collected through face-to-face interviews. They suggested that it is better to use interviews to increase the validity of self-reported SHS exposure. Based on a literature review, we hypothesised that the household is an important factor influencing the accuracy of self-reported SHS exposure among non-smoking pregnant women. This study aimed to compare self-reported SHS exposure to urinary cotinine levels.

## 2. Materials and Methods

### 2.1. Study Design and Sampling

A cross-sectional, correlational design was used to examine the correlates of objective exposure to SHS. This study involved data collection from October 2017 to February 2019. This study used structured questionnaires with a convenience sample of 70 participants at a hospital in Midwest Taiwan. One hundred and six potential participants were invited to participate in the study conducted in a waiting room of the maternal outpatient department (OPD) in a regional hospital. Seventy pregnant women met the inclusion criteria, whereas 36 declined or did not meet the inclusion criteria. The response rate was 66%. Potential participants were referred by a registered nurse in the maternal OPD. Thereafter, a researcher was approached to determine whether the participants met the inclusion criteria. Subsequently, one-on-one interviews were conducted to complete the self-reported SHS questionnaire. Urine samples were collected on the same day of the prenatal care visit. The inclusion criteria were as follows: (1) singleton pregnancy of 12–16 weeks, (2) age of 20–45 years, (3) ability to read Chinese and speak Mandarin or Taiwanese, and (4) residence in Chiayi for at least 6 months. We excluded women who smoked after pregnancy confirmation or had pregnancy-related complications, such as placenta previa, gestational diabetes mellitus, pregnancy-induced hypertension, cardiac disease, and premature rupture of membranes. A minimum of 68 participants were required to have 80% power to yield significant results, with a medium effect size of f2 = 0.15 and an alpha level of 0.05 [20].

### 2.2. Ethical and Research Approvals

This study was approved by the institutional review board. Potential participants were informed of its purposes, benefits, risks, and that their voluntary participation would remain anonymous and confidential. They were also reassured that they could terminate at any time. Those who agreed to participate provided written informed consent. The anonymity of the study participants was warranted using a study identification number without any personal identifiers.

### 2.3. Measurements

All instruments were obtained from self-reported questionnaires and laboratory confirmation of cotinine levels in urinary samples. Instruments and measures included the following: (1) a structured demographic questionnaire, (2) subjective exposure to smoke, a questionnaire regarding environmental tobacco smoke (SHS), and (3) objective exposure to smoke (cotinine concentration level).

#### 2.3.1. Demographic Characteristics

The demographic questionnaire included personal details, including age, weight, height, education level, employment, and religion. 

#### 2.3.2. Subjective Exposure to Smoke

The self-reported SHS exposure questionnaire was used to determine the participants’ exposure at home, workplace, and public places; instances of smoking in front of participants (yes); the number of smokers at home; SHS exposure in the previous 7 days; and their perceived frequency of SHS exposure in the previous 7 days, respectively. To assess SHS exposure in the past 7 days, participants were selected from seven potential exposure sources, including coffee shop or restaurants, car, their home and other houses, malls or shopping centres, at or near schools, and work [21,22,23]. Response choices for each source were never, once, a few times, more than a few times, and a lot. Responses for this measure ranged from 0 to 28, with higher scores indicating greater exposure. The perceived frequency of SHS exposure was based on a one-item scale that assessed the degree of perceived frequency of exposure to SHS on a scale of 0 to 10 [23,24]. Higher scores indicated greater perceived exposure.

#### 2.3.3. Objective Exposure to Smoke 

Nicotine is a common smoking biomarker. Cotinine is a nicotine metabolite, and approximately 70–80% of absorbed nicotine is converted into cotinine [25]. In addition, it has a higher sensitivity and specificity than nicotine [25]. Urinary cotinine has a relatively longer half-life (16–20 h) than nicotine [26]; however, the half-life of cotinine is much shorter (16.6 h) for pregnancy [27]. Urinary cotinine is often used to assess SHS exposure because it is non-invasive, and the average half-life of cotinine in the serum, urine, and saliva is nearly similar [16,28,29]. A urine cotinine concentration of 100 ng/mL was used to identify smokers in the general population [30,31]. Passive smokers and non-smokers showed urinary cotinine levels of 40–100 ng/mL and <40 ng/mL, respectively [31]. However, the clearance of nicotine and cotinine is significantly higher, and the half-life of cotinine is much shorter during pregnancy [32]. Based on the study by Aurrekoetxea et al. [33], the cut-off point for discriminating occasional smokers from non-smokers was 27 ng/mL (95% confidence interval (CI), 11–43), and the cut-off point for differentiating non-SHS-exposed from non-smokers was 19 ng/mL (95% CI, 11–33). In this study, the content of cotinine in human urine was ascertained using ultra-performance liquid chromatography-tandem mass spectrometry following the method described by Xu et al. [34]. 

#### 2.3.4. Statistical Analysis

**Figure 1:**
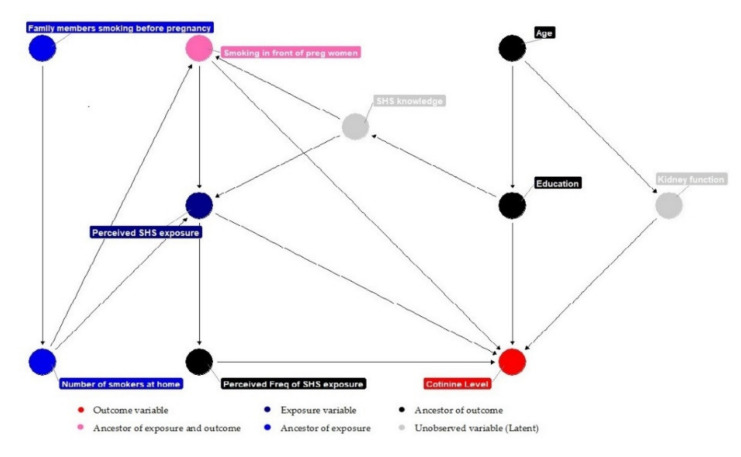
Directed acyclic graph representing the causal assumptions used for covariate selection.

Data were analysed using frequency distributions and descriptive statistics. Before performing regression analyses, *t*-tests, Pearson’s correlations, or Spearman rank correlation were used to analyse the relationships among the variables. A two-step model-building procedure was used to determine variables to assess the correlates of subjective to objective SHS exposure (urinary cotinine levels) in a multivariate regression model. In the first step, the variable selection was based on a causal path diagram created using R programs using the directed acyclic graph (DAG) approach [35]. Covariates were selected based on the recent literature [27,36,37,38], known correlates for SHS exposure in pregnant women, and hypothesised relationships. The body mass index (BMI) was not included in the statistical model for two reasons: (1) Baltar et al. found that the BMI was inversely associated with cotinine concentration levels; however, this relationship was not observed in non-smokers [36]; (2) There is no strong evidence that the BMI causally influences cotinine levels [38]. Using the DAG approach, a priori model of the postulated relationships between SHS exposure, urinary cotinine levels, and covariates was established. By adjusting for these factors, the effect of all the described covariates in Figure 1 on the urinary cotinine levels was investigated. Thereafter, univariate analyses were conducted to determine the unadjusted associations between subjective and objective SHS exposure and potential correlates. In the second step, as recommended by Hosmer et al. [39], only the variables associated with urinary cotinine levels (*p* < 0.25) were included in the multivariate model. Statistical significance was set at *p* < 0.05. Data analyses were conducted using SPSS 25 (IBM SPSS Inc., Chicago, IL, USA).

## 3. Results

### 3.1. Characteristics of the Participants

The descriptive characteristics of the participants are presented in Table 1. This study included 70 women. The mean age was 32.0 years (standard deviation (SD) = 4.0 years) with an average education level of 17.9 years (SD = 2.8 years). The mean body weight was 60.7 kg, and the BMI was 23.5 kg/m^2^. Approximately half (n = 34, 48.6%) of the family members of the study participants smoked before their pregnancy. In contrast, only a few (*n* = 5, 7.5%) of the study participants’ partners and family members smoked in front of the pregnant women. The average urinary cotinine level was 6.8± 6.3 ng/mL and ranged from 1.0 to 41.1 ng/mL (Table 1).

Regarding the perceived frequency of SHS exposure in the past 7 days, the participants reported that most of them did not perceive SHS exposure either at home, at work, or in the car (*n* = 46, 67.6% vs. *n* = 41, 61.2% vs. *n* = 51, 77.3%). However, they were exposed to SHS for 1 to 2 days in restaurant or coffee shop or shopping centre (*n* = 37, 55.2% vs. *n* = 34, 50.0%). Additional information regarding the perceived frequency of SHS is shown in Table 2.

### 3.2. Relationships between Exposure Variables of Interest and Urinary Cotinine Level

The results of multiple regression analyses are presented in Table 3. Based on the several studies [36,38], the DAG approach and the correlation among the BMI, perceived SHS exposure in the 7 past day, and the cotinine levels (r = −0.07 vs. r = 0.000, respectively), it was excluded in the model. The correlation between urinary cotinine and correlates in the univariate regression analyses were age, education (years), family members smoking before pregnancy, the number of smokers at home, perceived SHS exposure in the past 7 days, perceived frequency of SHS exposure in the past 7 days, and smoking in front of the participants. However, in the multivariate model, only smoking in front of the participants and perceived frequency of SHS exposure in the past 7 days were significantly associated with cotinine levels after controlling for potential confounders (F = 4.8, *p* = 0.002; Table 3). 

## 4. Discussion

The median of urinary cotinine level was 6.0 ng/mL and ranged from 1.0 to 41.1 ng/mL. This result is consistent with the characteristics of participants who did not smoke and were exposed to SHS. Studies have shown that, although nicotine can be absorbed by SHS, the levels absorbed are insufficient to increase urine cotinine levels to greater than 500 ng/mL [40]. In addition, during pregnancy, blood and urinary cotinine levels are often reduced due to increased renal clearance [32]. Our results regarding the range of the urinary cotinine levels (1.0-41.1 ng/mL) are consistent with Aurrekoetxea et al. [33], who reported the urine cotinine levels in the pregnant women exposed to SHS were above 19 ng/mL, depending on the duration and sources of SHS exposure.

This study is the first in Taiwan to validate measurements of psychosocial SHS exposure and urinary cotinine biomarkers. The results showed that two questions from the questionnaire were significantly associated with cotinine biomarkers among pregnant women (Table 3). This study adds to the body of knowledge of SHS exposure using a self-reported method in a cohort of pregnant women [17,18,41,42,43]. Within the context of this study, it is a Chinese social norm to avoid causing any harm during pregnancy, including SHS exposure. Therefore, a strong awareness of avoiding harm may protect Taiwanese pregnant women from SHS exposure. This may explain why a small portion of participants reported SHS exposure in our study and demonstrated reliable evidence of self-reported SHS exposure. Our results are similar to those of Govarkovs et al.’s study [18], who reported that women’s avoidance of SHS exposure is consistent with cotinine levels. In this study, the question item ’instances of smoking in front of the participant’ was significantly associated with urinary biomarkers (β = 0.6, *p* < 0.001; Table 3). In addition, the perceived frequency of SHS exposure in the past 7 days (β = 0.3, *p* = 0.004; Table 3) was the most associated corelate, second only to ‘instances of smoking in front of the participants’. Therefore, the proposed self-reported questions, ‘instances of smoking in front of the participant’ and ‘subjective perceived frequency of SHS exposure in past 7 days’ may be suitable, with relative confidence, to assess the SHS exposure in pregnant women when objective measures are unavailable. 

The results of this study are consistent with those of Okoli [16]. In the United States, Okoli [16] surveyed 20 non-smoking adults to establish associations between various biomarkers of SHS exposure and self-reported SHS survey questionnaires. Okoli [16] found that hair nicotine yielded a moderate strength of association with the perceived frequency of SHS exposure. Okoli’s study is further supported by Martinez-Sánchez et al.’s study [44], who conducted a cross-sectional study involving 49 non-smoking volunteers to validate airborne nicotine, urinary cotinine, and self-reported questionnaires of SHS exposure in Spain. The survey questionnaire comprised two questions to gather information regarding SHS exposure in the home setting: (1) the perception of the intensity of exposure to SHS in the last 7 days based on a Likert scale based on high, medium, low, and very low response; (2) to indicate a scale from zero to 10 about the perception of intensity of SHS exposure. Martinez-Sánchez et al. found a positive relationship between the perception of SHS and each direct SHS measure [44]. Moreover, a high correlation of scores with airborne nicotine (r = 0.86, *p* < 0.05), salivary cotinine (r = 0.75, *p* < 0.05), and urinary cotinine (r = 0.62, *p* < 0.05) was reported. This study supports that selected self-report questions may be useful to assess SHS exposure at home.

Individuals’ homes may be a major place for SHS exposure for women and children despite public education on reducing tobacco smoke exposure in homes [42,45,46]. Household members are still considered a strong predictor of SHS exposure among women and children [2,45,47,48]. In a cohort of 18,845 Taiwanese children aged 18 months, it was found that 62% of these children lived in a household with at least one smoker [48]. Cheng et al. [48] further found that 84% of the smokers in the household were fathers of children. Consistently, in Egypt, Abdelati et al. [45] also found that more than 50% of 118 pregnant women were exposed to SHS by their spouses. In Japan, a study aimed at ascertaining the validity of SHS exposure and plasma cotinine found that 63% of non-smoking pregnant women were exposed to SHS [42]. According to the Health Promotion Administration, Ministry of Health and Welfare (Taiwan) [46], campaigns to reduce smoking in the workplace and public led to a gradual decline in the prevalence of smoking Taiwan. However, in this study, in contrast to individuals’ homes, restaurants/coffee shops or shopping centres/convenience stores have the highest SHS (Table 2). Smoking in public and in the workplace remains an issue [49]. Thus, those who stay for long durations at home and in public places are at increased risk of SHS hazards. Therefore, reducing SHS exposure at home and in public places remains an important challenge to be tackled. It is recommended that the efforts of tobacco ban policy in public places should be maintained.

This study has some limitations that should be acknowledged. First, the small sample size (*n* = 70) may increase the risk of type II errors, restricting the generalizability of the findings. However, our sample size was sufficient for the study to have 80% power to yield significant results (*n* = 68), with a medium effect size of f2 = 0.15 and an alpha level of 0.05 [20]. Second, we only recruited and examined pregnant women in an outpatient clinic or maternity care. Thus, the results might have potential selection bias and may not generalise the national population of pregnant women in Taiwan. However, our population characteristics are representative of the Taiwanese national profile specified in the 2016 Family and Fertility Survey Report [50], which might have decreased the bias of the single-centre study. In addition, sampling times could have impacted the urinary cotinine level; therefore, the different urine sampling times may have been biased because of the unknown time interval between reporting of SHS and urinary sampling. Finally, our study did not collect hair to examine hair nicotine to detect the chronic influence of SHS over a longer duration. The urinary cotinine level in pregnant women only represents an acute-moderate impact of SHS within one day. Further studies may be needed using hair samples to identify the association between long-term SHS exposure and self-report questionnaires. 

## 5. Conclusions

This study provides additional data in the existing literature by investigating the associations between metabolic components of SHS in urine and survey SHS exposure measures to assess the representative items of SHS exposure among pregnant women. Questions regarding ‘instances of smoking in front of the individual’ appears to be a feasible question for use in pregnant women in clinics when assessing SHS exposure. Hence, we suggest that this simple question should be included and monitored in clinical assessments to support pregnant women in reducing the hazards associated with SHS exposure. 

## Figures and Tables

**Table 1:** Characteristics of pregnant women in this study (*N* = 70).

Characteristics	Subjects N	*N* (%) or Mean ± Standard Deviation	Range
**Demographic characteristics of participants**			
Age, year	70	32.0 ± 4.0	24.0–44.0
Weight, kg	66	60.7 ± 11.2	40.2–95.0
Body mass index, kg/m^2^	66	23.5 ± 3.9	17.6–35.9
Education level, year	70	17.9 ± 2.8	12.0–21.0
Family members smoke before pregnancy (yes)	70	34 (48.6%)	–
**Subjective exposure to smoke**			
Smoking in front of participants (yes)	67	5 (7.5%)	–
Number of smokers at home	70	1.0 ± 1.2	0.0–5.0
Perceived SHS exposure in past 7 days	64	3.2 ± 2.3	0.0–28.0
Perceived frequency of SHS exposure in past 7 days	69	2.4 ± 2.2	0.0–10.0
**Objective exposure to smoke**			
Level of UC, ng/mL	70	6.0 (Median)	1.0–41.1
UC Quartile, n (%)			
0–25%		21 (30.0%)	
26–50%		15 (21.4%)	
51–75%		17 (24.3%)	
76–100%		17 (24.3%)	

SHS = second-hand smoke; UC = urinary cotinine.

**Table 2:** Perceived frequency of SHS in multiple sites among pregnant women.

	Never	1 to 2 Days/Week	3 to 4 Days/Week	5 to 6 Days/Week	7 Days/Week
	*N* (%)	*N* (%)	*N* (%)	*N* (%)	*N* (%)
In restaurant/Coffee shop (*n* = 67)	28 (41.8)	37 (55.2)	2 (3.0)	0 (0.0)	0 (0.0)
Car (*n* = 66)	51 (77.3)	13 (19.7)	2 (3.0)	0 (0.0)	0 (0.0)
At home (*n* = 68)	46 (67.6)	18 (26.5)	3 (4.4)	0 (0.0)	1 (1.5)
In others house (*n* = 68)	43 (63.2)	24 (35.3)	1 (1.5)	0 (0.0)	0 (0.0)
In shopping centre/Convenience store (*n* = 68)	33 (48.5)	34 (50.0)	1 (1.5)	0 (0.0)	0 (0.0)
At/near school (*n* = 68)	49 (72.1)	17 (25.0)	2 (2.9)	0 (0.0)	0 (0.0)
At work (*n* = 67)	41 (61.2)	20 (29.8)	4 (6.0)	1 (1.5)	1 (1.5)

**Table 3:** Univariate and multiple regression analysis of the factors associated with urinary cotinine levels of pregnant women.

Urinary Cotinine Level	Univariate/Unadjusted		Multiple/Adjusted	
ng/mL	*Β* (95% CI)	*p*-Value	*Β* (95% CI)	*p*-Value
Age/y	−1.1 (−4.3 to 2.1)	0.5	−	−
Education/y	−5.2 (−9.7 to 0.7) *	0.025 *	−0.7 (−0.7 to 0.3)	0.5
Family members smoke before pregnancy	−12.8 (−37.9 to 12.2)	0.3	−	−
Smoking in front of participants	179.2 (39.5 to 118.9) **	0.00 **	0.6 (8.1 to 18.4) ***	0.000 ***
Number of smokers at home	8.7 (−1.8 to 19.2)	0.1	−0.03 (−1.6 to 1.2)	0.8
Perceived SHS exposure in past 7 days	2.4 (−3.8 to 8.5)	0. 5	−	−
Perceived frequency of SHS exposure in past 7 days	6.1 (−2.1 to 14.3)	0.1	0.3 (0.3 to 1.6) **	0.004 **

Seven variables were analysed by univariate analysis and four variables that were associated with urinary cotinine levels (*p* < 0.25 were included in the multivariate model, B = unstandardized regression coefficient; CI = confidence interval. * *p* < 0.05, ** *p* < 0.01, and *** *p* < 0.001.

## Data Availability

The data presented in this study are available on request from the corresponding author. The data are not publicly available due to the privacy of research participants.
